# Lattice filter for processing image data of three-dimensional protein nanocrystals

**DOI:** 10.1107/S205979831502149X

**Published:** 2016-01-01

**Authors:** E. van Genderen, Y.-W. Li, I. Nederlof, J. P. Abrahams

**Affiliations:** a Biophysical Structural Chemistry, Leiden University, Einsteinweg 55, 2333 CC Leiden, The Netherlands; b Amsterdam Scientific Instruments, Postbus 41882, 1009 DB Amsterdam, The Netherlands

**Keywords:** nanocrystals, lattice filter, cryo-EM

## Abstract

When 300 kV cryo-EM images at Scherzer focus are acquired from ∼100 nm thick three-dimensional protein nanocrystals using a Falcon 2 direct electron detector, Fourier transformation can reveal the crystalline lattice to surprisingly high resolutions, even though the images themselves seem to be devoid of any contrast. Here, it is reported how this lattice information can be enhanced by means of a wave finder in combination with Wiener-type maximum-likelihood filtering. This procedure paves the way towards full three-dimensional structure determination at high resolution for protein crystals.

## Abstract 2

A specialized filter for finding lattices in images of three-dimensional nanocrystals devoid of any contrast is described.

## 1. Introduction   

Previously, we demonstrated that electron diffraction of protein three-dimensional nanocrystals could yield 2 Å resolution data (Nederlof, van Genderen *et al.*, 2013 ▸). Recently, we collected hundreds of high-resolution electron images at Scherzer focus from cryopreserved, randomly orientated three-dimensional nanocrystals of our test protein lysozyme (Nederlof, Li *et al.*, 2013 ▸). Although appearing to be devoid of signal, Fourier transformation revealed crystalline order to a resolution of 4 Å or better in about 50% of cases. Crystals with a thickness of about 100 nm (corresponding to 15–30 unit cells) yielded data with the best quality. The resolution of the Bragg spots in the Fourier transform of the electron micrograph is the lower threshold of the crystalline order. If the crystal occupies only part of the electron image, the remainder of the image contributes nothing but noise. If the crystal is cracked, twinned, warped or contains mosaic blocks, the resolution of the Fourier transform is reduced because the unit cells do not align perfectly. In two-dimensional crystallo­graphy the resolution is enhanced by computationally ‘unbending’ the crystal (Gil *et al.*, 2006 ▸; Henderson *et al.*, 1990 ▸; Kühlbrandt & Wang, 1991 ▸). Firstly, the two-dimensional lattice repeat is identified and the frequencies that do not conform to this repeat are filtered from the Fourier transform of the image by setting them to zero, thus enhancing the translationally repeating features of the image. This is equivalent to averaging the image with shifted versions of itself, whereby the magnitude and direction of the shifts are determined by the lattice parameters. This procedure will therefore average out noise that does not have translational symmetry, as it is not correlated to the signal. The image of the crystal is then subdivided into patches, which are subsequently aligned and averaged (Zeng *et al.*, 2012 ▸; Stahlberg *et al.*, 2001 ▸; Scherer *et al.*, 2014 ▸).

One of the main differences between two-dimensional crystals and three-dimensional crystals is that projection images of randomly oriented three-dimensional crystals usually show moiré patterns, rather than regular two-dimensional lattices. Hence, it is not possible in the general case to extract a repeating unit: the moiré is not usually defined as a rational sum of the other two independent lattice vectors. The moiré pattern (a potentially nonrepeating pattern that results from the superposition of multiple lattices) exists because of the three independent lattice parameters characterizing the three-dimensional crystal. Owing to Ewald sphere curvature, beam divergence and/or crystal mosaicity, repeats corresponding to each of these three translational symmetries can co-exist in one and the same image. If one of the translation operators cannot be expressed as an integer sum of the other two, a moiré pattern results. While this pattern does not directly show the crystal lattice, it does contain information on this lattice.

Here, we discuss a procedure for enhancing the moiré lattice information in the analysis of three-dimensional nanocrystals that does not require knowledge of the lattice parameters or orientation of the crystal, and even allows the the lattices to be enhanced if multiple crystals are present in the image.

A high-resolution image of a (three-dimensional) crystal will have translational symmetry. Although this symmetry may not be obvious because of noise, the amplitudes of a Fourier transformation of the image will reveal the reciprocal lattice. One way of enhancing the translational symmetry in the image therefore is to identify the parameters that describe the recip­rocal lattice, then to zero all reciprocal pixels that do not belong to this lattice and reverse the Fourier transform as in two-dimensional crystallography. However, this approach has a mathematical flaw, which can be understood intuitively as follows. Suppose a high background and a weak Bragg spot in the Fourier transform of the image. If all reciprocal pixels are set to zero but the Bragg pixels are kept at their original value, then this will lead to an incorrect estimation of the relative strength of this particular Bragg spot. This is equivalent to overweighting a weak Bragg spot relative to a stronger Bragg spot that is higher above the background. The Wiener filter addresses this problem in a maximum-likelihood approach. An improvement over existing methods is obtained by assuming that the power spectrum of the noise and the lattice signal are uncorrelated. This is equivalent to establishing a Wiener filter that optimally enhances the lattice. Our approach does not need knowledge of the crystal lattice constants nor of the crystal orientation or location to obtain a good result.

**Figure 1:**
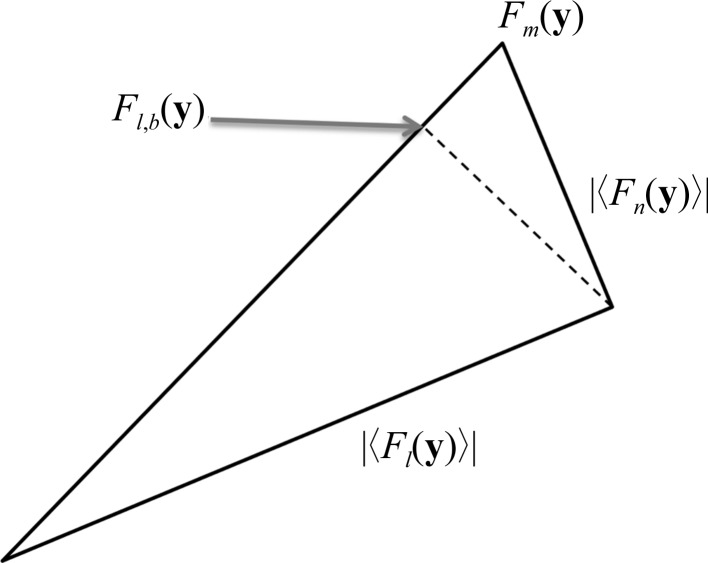
The measured structure factor *F_m_*(**y**) is the sum of the unknown structure factor corresponding to the lattice signal *F_l_*(**y**) and the unknown structure factor corresponding to the noise *F_n_*(**y**). On average, *F_l_*(**y**) and *F_n_*(**y**) are uncorrelated; hence, the expected absolute phase difference between them is π/2. We can only infer the absolute values of the expected structure factors. We cannot infer their phases because there are two equally valid solutions mirrored over *F_m_*(**y**). The best estimate for *F_l_*(**y**) is denoted by *F*_*l*,*b*_(**y**). It can be calculated by projecting 〈*F_l_*(**y**)〉 onto *F_m_*(**y**).

Assume a structure factor of the Fourier transform of the image: *F_m_*(**y**). It is the sum of the structure factor of the crystal lattice *F_l_*(**y**) and the structure factor of the noise *F_n_*(**y**), 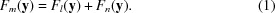



Neither *F_l_*(**y**) nor *F_n_*(**y**) are known. We can only assume that they are uncorrelated. Their expected absolute phase difference will therefore be π/2; hence, together with *F_m_*(**y**) they define a right-angled triangle, 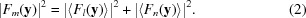



We can infer |〈*F_n_*(**y**)|〉^2^ from the power spectrum of the image and use the result to calculate the expected amplitude of *F_l_*(**y**). However, we also require its phase, and the only reasonable estimate is the phase of *F_m_*(**y**). Therefore, we need to project 〈*F_l_*(**y**)〉 onto *F_m_*(**y**) to obtain the best estimate of the expected lattice structure factor *F*_*l*,*b*_(**y**) (Fig. 1 ▸).

Geometry implies the following equality, which is equivalent to an optimal (Wiener) filter (Press *et al.*, 2007 ▸), 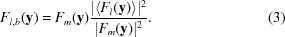



Substituting with (1) gives
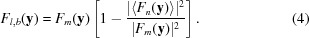



Thus, scaling the structure factors of the original image by this likelihood will recover the phases of the waves as well as their amplitudes. This theory leads to a more robust algorithm as described below.

## 2. Method   

**Figure 2:**
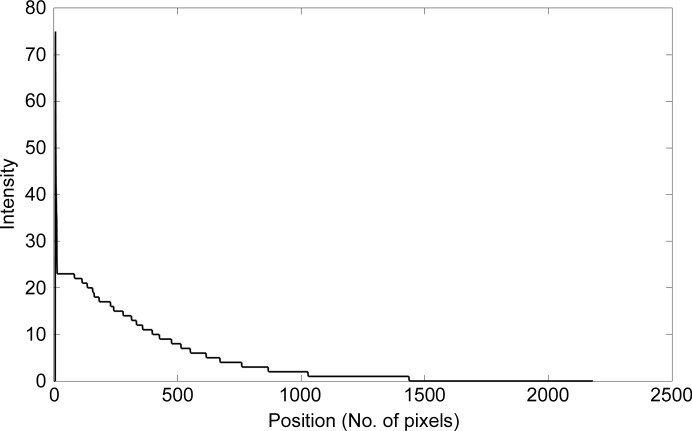
Plot of the rotational average of Fig. 3 ▸(*c*).

**Figure 3:**
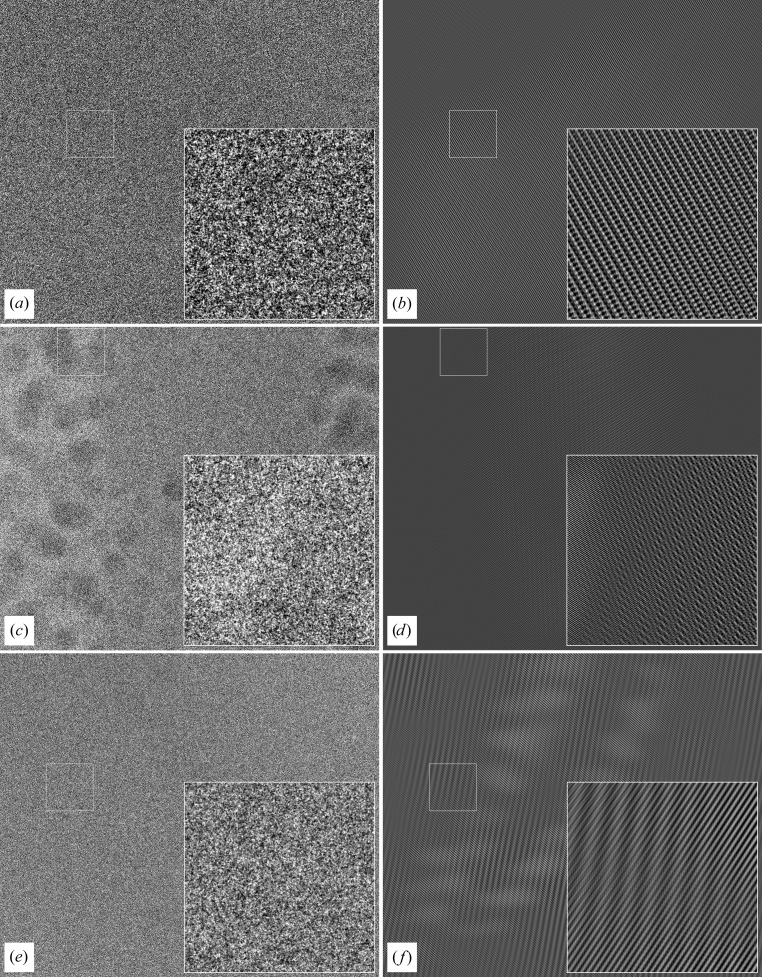
Images of 100 nm thick three-dimensional protein crystals produced by a Titan Krios 300 kV FEG transmission EM and captured with a Falcon 2 camera (4096 × 4096 pixels) using an 0.5 s exposure time and an illumination of 3–10 e^−^ Å^−2^. Left, original unprocessed images; right, processed images. (*a*, *b*) Lysozyme crystal; (*c*, *d*) lysozyme crystal with several ice crystals included (shown in detail); (*e*, *f*) crystal of a cross-β peptide. Notice that in the cutout of the results after filtering of the middle image (*d*) we can distinguish an ice crystal (with a small unit cell and dominant spacings at ±3.8 Å at the left side of the cutout) from the protein crystal (right side of the cutout).

In order to prevent wrap-around artifacts, we padded the images with pixel values which were set to the average value of the original image. The amount of padding can be defined by the user and corresponds to the expected size of the crystalline domains. The default value (used throughout the paper) is 1/16th of the image size (corresponding to 256 pixels for Falcon 2 images). The pixels of the Fourier transform *F_m_*(**y**) of an electron image *I*(**x**) = 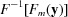 contain complex numbers (hence they carry phase information). Firstly, we calculate the radially averaged power spectrum |*F_n_*(|**y**|)|^2^ of the image in order to approximate the power spectrum of the noise, 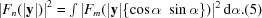
The radial average (2) is not completely smooth because of the contributions of the spots at certain spacings. In order to correct for this, we assumed that the radial average of the ‘noise’ power spectrum is a decreasing function of |**y**|. Thus, if *F_n_*(**y**) increases, this must be caused by the signal of Bragg spots. In this case, we keep *F_n_*(**y**) constant until is decreases below this value. An example of such a radial average is shown as a linear plot in Fig. 2 ▸; this is the result from the image in Fig. 3 ▸(*a*). Then, for each pixel, we calculate its significance *s*(**y**) as a normalized signal-to-noise ratio, 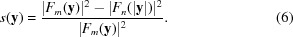
In the absence of noise [*s*(**y**) = 1] and when the norm^1^ of the signal is equal to the norm of the noise, [*s*(**y**) = 0]. Note that *s*(**y**) can be negative owing to fluctuations in the noise level. In fact, in the absence of signal, fluctuations in the noise level will cause *s*(**y**) = 1 to be negative for half of the reciprocal pixels! We consider a pixel to contribute significant information about the lattice when *s*(**y**) is higher than a specified cutoff value. Pixels lower than the cutoff value are then set to zero. As a default, we used a cutoff value (*c* = 0.0) for all of the examples in this paper. Thus, for all examples given, all pixels of the lattice filter *L*(**y**) which had a norm below the radially averaged norm of *F*(**y**) were set to zero. The value of the remaining pixels of the lattice filter *L*(**y**) were set to
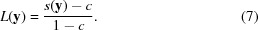



This lattice filter *L*(**y**) can be still be noisy, especially if the signal is low, so we included the option of only considering pixels that are likely to belong to a Bragg spot. The lattice parameters of the image are usually unknown at this stage of data analysis. Thus, to identify potential Bragg spots, we used a method that does not require lattice parameters. Firstly, we selected 3 × 3 clusters of pixels in which each of the pixels had a norm that is above a specified acceptance level *a*. We only allowed pixels to have nonzero values if they are less then a specified distance *r* away from any pixels within clusters which represent spots. This cutoff distance *r* is proportional to the reciprocal-space equivalent of the expected size of the crystalline domains. All pixels of *L*(**y**) that are further away than *r* pixels from such a cluster of significant pixels [for which *L*(**y**) > *a*] were set to zero. As defaults, we used an acceptance value of *a* = 0.4 and a Bragg spot radius of *r* = 4 pixels for all of the examples in this paper. This distance criterion can be suppressed by setting *a* = *c*.

In addition to the lattice, this procedure also enhances other repetitive features of the image. Detector artifacts in particular can be a major source of such spurious features. We found these artifacts to produce high-resolution features. We therefore included an option to filter out such artificial signals by setting *L*(**y**) to zero for all |**y**| > *n*|**y**|_max_. As a default value we used a Nyquist cutoff of *n* = 2/3 for all examples in this paper.

After having constructed the lattice filter *L*(**y**) according to the procedure outlined above, we calculated the filtered image *I_f_*(**x**), 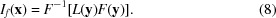



The nanoprotein crystal images used here as an illustration of the method were acquired on an FEI Titan Krios electron microscope at Scherzer focus from crystals with a thickness of approximately 100 nm. The data were collected using a Falcon 2 FEI camera on 4048 × 4048 pixels with 0.5 s exposure time. The mean dose of the exposures was 3 to 10 e^−^ Å^−2^ (for further details, see Nederlof, Li *et al.*, 2013 ▸).

## 3. Results   

Three example images (see Fig. 3 ▸) from lysozyme nanocrystals show the merits of our new algorithm.

The processed images show moiré patterns that are typical of non-oriented three-dimensional crystals. Owing to truncation errors, some spurious repeating features will also be visible in areas of the image where no crystal is present, but here the amplitudes are much lower than in the crystal. Thus, the processed images will give a clear indication of where the crystal might be located. The result of the filter algorithm is shown in Fig. 3 ▸. This information can then be used for further analysis as described in Nederlof, Li *et al.* (2013 ▸)

If the images are not padded as described in §[Sec sec2]2, a wrap-around effect will occur and the information tends to bleed over the edge of the image into the opposite side of the image. This can be circumvented by padding the image, but when this padding is removed after lattice filtering the resulting discontinuities at the image edges can lead to crosses centred on the Bragg spots in reciprocal space, which could be undesirable for certain applications. Crosses can be prevented by choosing not to pad the images, or they can be suppressed by writing out filtered images without removing their padding (not shown).

**Figure 4:**
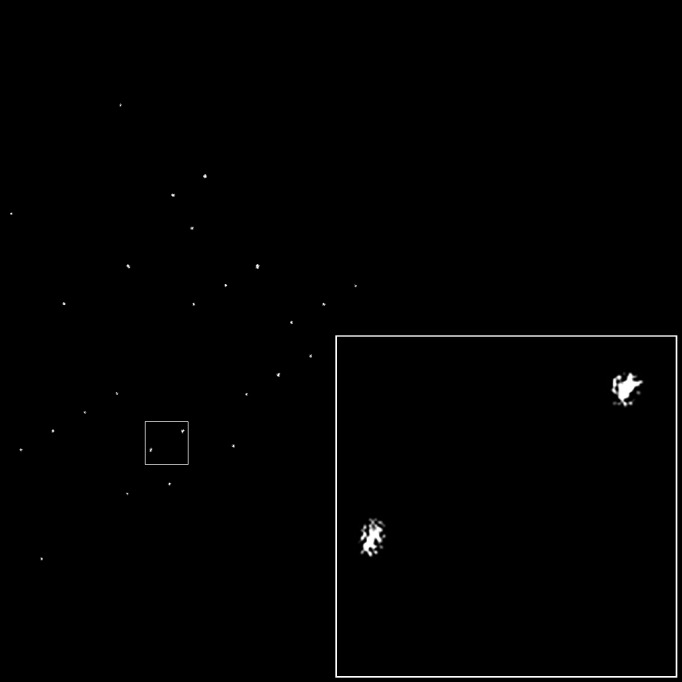
Unique half of the centrosymmetric lattice filter of the lysozyme crystal image from Fig. 3 ▸. The spots that make up the lattice of the real image are clearly visible. Because of mosaicity and crystal shape, the spots are not uniform in shape.

Close inspection of the power spectrum shows that the spots do not have a uniform shape (Fig. 4 ▸). The shapes of the spots differ when the projection of the crystal contains separate crystallites. The latter we call mosaicity and it shows that domains can exist within a crystal and can each have a slightly different orientation. While this does not change the meta­structure of the lattice, it will disrupt the moiré pattern and makes interpreting the structure more difficult. To put a positive twist on this, it also provides more orientations of the crystal.

**Figure 5:**
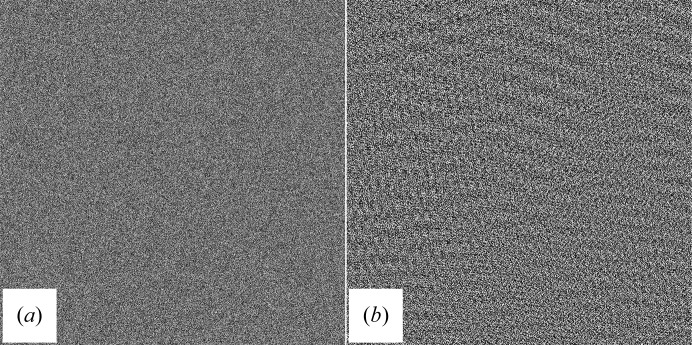
(*a*) Nonfiltered random-noise image with an intensity and standard deviation comparable to the image of a crystal in Fig. 3 ▸(*a*). (*b*) Filtered random-noise image.

How does our filter behave when applied to a image consisting of generated noise with the same median intensity and standard deviation as an image containing a crystal (values have been obtained from Fig. 3 ▸ *a*)? The result of such a filtering is shown in Fig. 5 ▸. While some weak lattice structures can be seen, the amplitude of the wave structures is only slightly above the median pixel level. This shows that the filter is able to discriminate between an image consisting of random noise and a low signal-to-noise ratio image which contains a crystal lattice.

**Figure 6:**
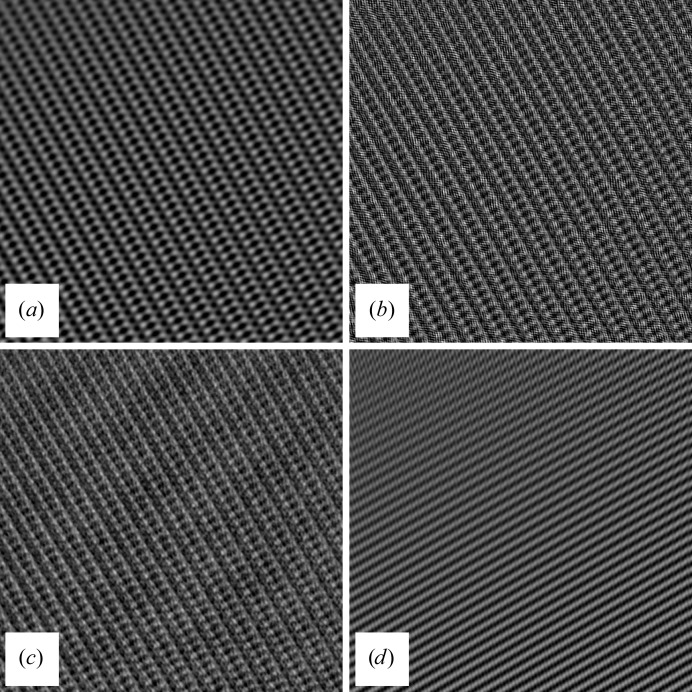
Results from the same area as in Fig. 3 ▸. (*a*) Image obtained by selecting only spots at a very low resolution. (*b*) Including the Nyquist frequency. The detector artifacts are visible as well as the effects of wrapping effects in reciprocal space. (*c*) Low spot-threshold selection; by including spots that are not Bragg spots more noise is present, but more detail in the lattice is also visible. (*d*) Very high spot-selection threshold, thereby selecting only the strongest spots.

The algorithm includes the possibility to set a resolution cutoff corresponding to a factor of the Nyquist frequency. While this is a powerful tool for removing certain detector artifacts or selecting a filter quality, misuse can introduce artifacts. Fig. 6 ▸ demonstrates the effect of the lattice filter when using a sub-optimal Nyquist cutoff. If it is too low, the high-resolution spots will be obliterated and therefore the high-resolution details of the crystal lattice will be lost. Choosing a value that is too high can result in a severe checkerboard striping and patterning owing to detector artifacts^2^.

The other important filter parameter is the spot-selection threshold. If a value is chosen above the noise level it will lead to the selection of bogus Bragg spots. This will introduce spurious noise in the filtered image. Another case is when the criteria for selecting the spots are too stringent and only a few of the brightest spots remain. In this case the final lattice image usually shows a one-dimensional or two-dimensional lattice pattern over the whole image, no longer discriminating between the crystal and its disordered surroundings.

## 4. Discussion   

In earlier work we reported interactive image processing to enhance the lattice. Although this produced good results, it was slow, tedious and required expert knowledge. Here, we show the mathematical proof of our new approach, captured in an automatic algorithm that is very fast (half a second for a 4096 × 4096 image on a standard 2014 desktop computer). The lattice filter is a very powerful tool for selecting and analyzing extremely low contrast cryo-images of three-dimensional protein/peptide nanocrystals. It confirms that the three-dimensional crystals are made up from multiple domains which are slightly differently oriented. Indeed, the algorithm can comfortably deal with multiple crystals with very different orientations, unit cells and/or space groups, as is witnessed in the middle panel of Fig. 3 ▸, which shows that the lattice of an ice crystal is enhanced just as well as the lattice of a protein crystal. Since more than two lattice parameters are required to describe the moiré lattice of a projected three-dimensional crystal, approaches from two-dimensional crystallography cannot be applied straightforwardly or without considerable reprogramming. While one can argue that patches of the crystal with different orientations should not be back-transformed together, it is something that can be performed directly after the filtering process (Nederlof, Li *et al.*, 2013 ▸).

Our method does not correct for the contrast-transfer function (CTF), but since it does not affect the phases of the projection image, a CTF correction can be performed after lattice filtering. Although in principle a CTF correction could precede the lattice filter, it advisable to first perform the filtering, since this also takes care of background removal. In the examples that we give here we did not perform any CTF corrections, as the data were collected at Scherzer focus, where the first sign reversal of the CTF occurs beyond the resolution limit of our images.

We propose the new lattice filter as a powerful tool for processing very noisy images with crystal structure factors (and thus with phase information) hidden within them. The filter is able to discriminate between noise images and the very noisy images with very low contrast which contain crystal-like structures. The lattice filter retains the shape of the spots in Fourier space and also retains any phase gradients within the Bragg spots (which determine the domain structure within the crystal). Thus, it retains all of the significant information from the Bragg spots. This will open the way to combining the phases acquired from stationary, two-dimensional images with intensities of rotation diffraction data taken from the same type of crystals. In this way, we expect to be able to phase the diffraction information of protein and peptide crystals.

## Footnotes

1 The real-valued norm of a complex value is the square of its absolute value: |*a* + *bi*|^2^ = *a* ^2^ + *b* ^2^.

2 The artifacts introduced by a detector have structure-like features and will be of major influence in the final filtered image, and should be taken into account. In the case of the Falcon 2 camera used for the showcase sample images, this can include chip-to-chip fluctuations and read-out gain reference artifacts. The latter effects are most clearly visible at the Nyquist frequency in the power spectrum of the image. The Nyquist frequency artifact reveals itself as a bright line of pixels around the edge of the power spectrum and a checkerboard or striped patterning over the whole filtered image. .
